# Anti-TNF Therapy Reduces Serum Levels of Chemerin in Rheumatoid Arthritis: A New Mechanism by Which Anti-TNF Might Reduce Inflammation

**DOI:** 10.1371/journal.pone.0057802

**Published:** 2013-02-27

**Authors:** Marieke M. J. Herenius, Ana S. F. Oliveira, Carla A. Wijbrandts, Daniëlle M. Gerlag, Paul P. Tak, Maria C. Lebre

**Affiliations:** 1 Department of Clinical Immunology and Rheumatology, Academic Medical Center, University of Amsterdam, Amsterdam, The Netherlands; 2 Department of Experimental Immunology, Academic Medical Center, University of Amsterdam, Amsterdam, The Netherlands; University Hospital Jena, Germany

## Abstract

**Background:**

Chemerin is a specific chemoattractant for macrophages and dendritic cells (DC). In addition, it can rapidly stimulate macrophage adhesion to extracellular matrix proteins and adhesion molecules and is able to activate fibroblast-like synoviocytes (FLS), suggesting a role in the pathogenesis of rheumatoid arthritis (RA). Chemerin is also an adipocytokine that has been related to the inflammatory state of endothelial cells and as such could be involved in the changes in endothelial cells in RA and perhaps increased cardiovascular morbidity. We investigated whether anti-Tumor Necrosis Factor (TNF) treatment affects chemerin levels.

**Materials and Methods:**

49 patients with active RA (disease activity score evaluated in 28 joints (DAS28) ≥3.2) were started on adalimumab therapy. Blood was drawn from patients while fasting at baseline and 16 weeks after initiation of treatment. Chemerin serum levels were measured by ELISA and related to disease activity, mediators of inflammation and known risk factors for cardiovascular disease.

**Results:**

Adalimumab therapy reduced chemerin serum levels, which was correlated with the reduction in DAS28 (*r* = 0.37, *p* = 0.009). In addition, the decrease in chemerin serum levels after anti-TNF treatment was associated with the decrease in serum levels of IL-6 (*r* = 0.39, *p* = 0.033) and macrophage migration inhibitory factor (MIF) (*r* = 0.31, *p* = 0.049). Baseline chemerin serum levels were not related to traditional risk factors for atherosclerosis, except perhaps for smoking (p = 0.07).

**Conclusions:**

This exploratory study shows that adalimumab therapy lowers chemerin levels, which is associated with the reduction in disease activity parameters, and inflammatory mediators IL-6 and MIF. This suggests a possible involvement of chemerin in the migration/retention of macrophages in the synovium.

**Trial Registration Nederlands Trial Register:**

NTR 857

## Introduction

The synovium in rheumatoid arthritis (RA) is characterized by a dense infiltrate, consisting of T and B cells, plasma cells, macrophages, dendritic cells (DC), and other cells. [1] Inflammatory chemokines present in the synovium contribute to the migration and/or retention of these immune cells.[2]–[4] Chemerin is a recently discovered chemokine that specifically modulates chemotaxis and activation of macrophages and DC (in particular plasmacytoid DC (pDC) and monocyte-derived DC). [5] Furthermore, it can contribute to inflammation by stimulating macrophage adhesion to extracellular matrix proteins and adhesion molecules. [6] The expression of chemerin receptor chemokine-like receptor 1 (CMKLR1) or ChemR23 on antigen-presenting cells (macrophages and DC) suggests that chemerin constitutes an important link between innate and adaptive immunity. Both the innate and adaptive immune responses are implicated in the pathogenesis of RA and might even occur in a parallel fashion. Related to this it was recently reported [7] in RA that endothelial cells and synovial lining and sublining cells express chemerin while its receptor ChemR23 was expressed on macrophages, immature DCs and fibroblast-like synoviocytes (FLS). Interestingly, chemerin induced the release of IL-6, chemokine (C-C-motif) ligand 2 (CCL2) and matrix metalloproteinase-3 (MMP-3) by RA FLS. [7] These data suggest that blockade of chemerin represents an attractive candidate for future drug development as it could disrupt disease perpetuation. Interestingly, chemerin has also been implicated in the pathogenesis of another immune-mediated inflammatory disease, psoriasis. Chemerin expression in psoriatic skin lesions preceded and paralleled accumulation of pDC and clinical expression of psoriasis. [8].

Chemerin also belongs to the novel adipocytokine family together with omentin, visfatin, nesfatin and vaspin. [9] As such, it has been associated with the metabolic syndrome, which is defined by dyslipidemia, abdominal obesity, elevated blood pressure, elevated plasma glucose levels and a pro-inflammatory state promoting atherosclerotic cardiovascular disease. [10] In RA the prevalence of the metabolic syndrome is increased. [11] In addition, ChemR23 is expressed by vascular endothelial cells and it is regulated by pro-inflammatory cytokines, including TNF. [12] These observations may suggest the involvement of chemerin in the changes in the endothelial cells of RA patients and as such in increased vascular morbidity in RA patients.

In this exploratory study, we aimed to provide insight into the mechanism of action of anti-TNF therapy on synovial inflammation and vascular disease in RA, Therefore, we investigated the effects of adalimumab treatment on chemerin levels. Primary questions were the relationship between chemerin serum levels, DAS28, markers of inflammation (IL-6 and MIF) and traditional factors of atherosclerosis.

## Materials and Methods

### Patients and Clinical Assessments

The details of this cohort were previously described. [13] The study was performed according to the Declaration of Helsinki and approved by the medical ethics committee. All participants gave written informed consent. Patients (n = 49) were selected for the current study based on the availability of serum at baseline combined with standardized follow up data on the response to adalimumab treatment 16 weeks after the initiation of adalimumab. The protocol for this trial and information on patient enrollment are available as supporting information; see Checklist S1, Protocol S1 and Enrollment log S1. Briefly, all patients had an indication for the use of anti-TNF therapy according to the guidelines of the Dutch Society for Rheumatology, which is active disease status (DAS28≥3.2) despite previous treatment with at least 2 conventional disease-modifying anti-rheumatic drugs (DMARDs). All patients started adalimumab (40 mg subcutaneously every other week). Clinical response at week 16 was determined according to the European League Against Rheumatism (EULAR) criteria. [14] Patients with EULAR good and EULAR moderate response were considered responders. Primary study endpoints were the relationship between the change in chemerin serum levels and the change in DAS28, ESR and CRP, and the change parameters of inflammation (MIF and IL-6 serum levels). Secondary endpoints were the relation between chemerin serum levels at baseline and DAS28, ESR and CRP and traditional risk factors for atherosclerosis as assessed by recording medical history including cardiovascular events, body mass index (kg/m^2^), smoking (current smoking and ever smoking), current medication, hypertension, lipid profile ((TC, HDL, LDL, TG, Apo A-I, Apo B, Lp(a)) and diabetes. In addition, we assessed the relationship between the change in chemerin serum levels and the change in lipid profile.

### Lipid Profiles and Assessment of Radiological Damage

Blood was drawn from patients while fasting at baseline and 16 weeks after initiation of adalimumab treatment. Lipid levels were measured as described previously. [13] Radiographs of the hands and feet were obtained at baseline and were scored using the Sharp/van der Heijde score. [15].

### Serum Enzyme-linked Immunosorbent Assay (ELISA)

Chemerin serum levels were determined using a commercially available DuoSet ELISA Development kit (DY2324; R&D systems Inc, Minneapolis, MN). The serum levels of IL-6 and MIF were assessed as described previously. [13] As stated above, fasting serum samples were stored at −80°C until use.

### Statistical Analysis

When indicated, a paired t-test or the Wilcoxon signed ranks test was used to determine significant changes from baseline. Probability values <0.05 were considered statistically significant. The Mann Whitney U test was used to detect differences between groups (sex, prior cardiovascular events, anti-citrullinated peptide antibody (ACPA) and IgM-rheumatoid factor (RF)-status or users vs. non-users of non-steroidal anti-inflammatory drugs (NSAIDs), statins or anti-hypertensive drugs). Correlations were assessed with the Pearson product-moment or Spearman rank-order correlation coefficients, whichever was appropriate. The calculations were performed with SPSS 18.0 for Windows (SPSS, Chicago, IL). To avoid multiple testing comparisons for the primary objective were tested formally, (i.e. change in chemerin and its’ association with the change in DA28, ESR, CRP, IL-6 and MIF) while secondary comparisons were described.

## Results

### Patients and Clinical Response

The baseline patient characteristics are described in Table 1.The DAS28 score decreased significantly 16 weeks after initiation of adalimumab treatment from 5.4 (±1.1, mean±SD) to 3.6 (±1.2, mean±SD). According to EULAR response criteria 33%, 45%, and 22% of patients, respectively, achieved good, moderate or no response. There was no association between baseline chemerin serum levels and baseline Sharp van der Heijde score.

**Table 1:** Baseline characteristics.

**Demographics**	
Age (years)	50±14
Female (%)	39 (81)
**Baseline characteristics**	
Disease duration (months)	61 (32 - 142)
Sharp/van der Heijde score	6.6 (0 - 25.5)
Rheumatoid factor positive (%)	34 (71)
Anti-CCP positive (%)	33 (67)
DAS28	5.4±1.1
Use of corticsteroids (%)	13 (26)
Use of NSAIDs (%)	36 (71)
ESR (mm/hour)	20 (11 - 35)
C-reactive protein (mg/L)	7.7 (4.8 - 18.5)
**Cardiovascular characteristics**	
BMI kg/m2	26.7±6.0
Smokers, current (%)	11(22)
Diabetes mellitus type 2 (%)	4 (8)
Prior cardiovascular event (%)	6 (12)
Systolic blood pressure	132±16
Diastolic blood pressure	80±9
Plasma triglycerides level	1.0±0.5
Total cholesterol level	4.8±1.1
HDL cholesterol level	1.5±0.4
LDL cholesterol level	2.9±1.0
Statin use (%)	7 (14)

Mean values ± standard deviation (SD), median and interquartile range or percentages are shown. BMI =  body mass index, ESR = erythrocyte sedimentation rate.

### Anti-TNF Treatment Decreases Chemerin Serum Levels, which is Associated with the Decrease in Disease Activity, Serum Levels of IL-6 and Macrophage Migration Inhibitory Factor

**Figure 1:**
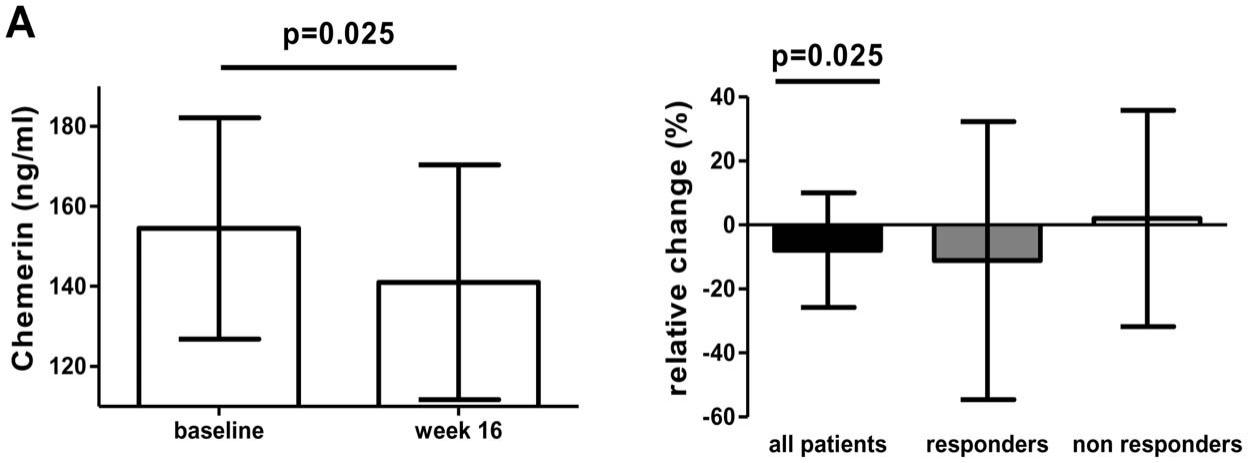
Changes in chemerin serum levels 16 weeks after initiation of treatment with adalimumab. (A) Absolute change in all patients (n = 49). Data are represented as mean values with standard deviation. (B) Relative change for all patients (n = 49), EULAR responders (n = 38) and EULAR non- responders (n = 11). Data are presented as mean values with standard deviation.

**Figure 2:**
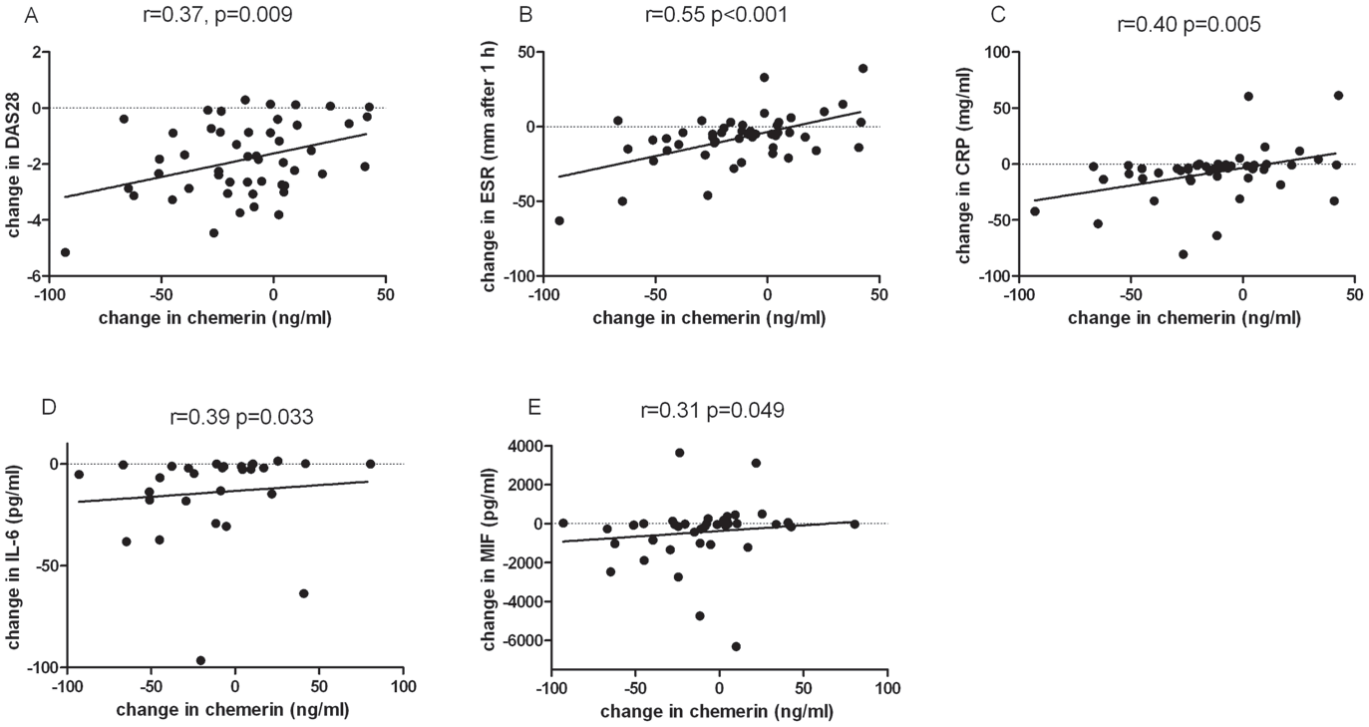
Changes in chemerin serum levels 16 weeks after initiation of treatment correlate with changes in disease activity parameters DAS28 (A, n = 49), ESR (B, n = 49) and CRP (C, n = 49) and changes in IL-6 (D, n = 29) and MIF (E, n = 41) serum levels, respectively.

At baseline chemerin serum levels varied from a minimum of 99 ng/ml to a maximum of 217 ng/ml. There was no relationship between chemerin serum levels at baseline and the use of low doses of corticosteroids, NSAIDs or the use of methotrexate (MTX). In addition, chemerin serum levels at baseline were not related to IgM-RF- or ACPA-status. Sixteen weeks after the initiation of adalimumab treatment chemerin levels showed a reduction from 154 ng/ml±28 ng/ml (mean±SD) to 141 ng/ml±29 ng/ml (mean±SD) (Figure 1A; p = 0.025). The decrease of chemerin serum levels 16 weeks after initiation of adalimumab treatment was seen only in EULAR responders (relative change −11.2±43.4 (mean±SD) and −11.3 [−30.3–3.9] (median, IQR)) and not in EULAR non-responders (relative change 2.0±33.8 (mean±SD) and 1.9%, [−23.3–33.7] (median, IQR) (Figure 1B). The decrease in chemerin serum levels was associated with decrease in parameters of disease activity DAS28 (*r* = 0.37, *p* = 0.009), ESR (*r* = 0.55 *p*<0.001) and CRP (*r* = 0.40, *p* = 0.005) (Fig. 2A–C, respectively). In addition, we found an association between the reduction in chemerin levels and the decrease in serum levels of IL-6 (*r* = 0.39, *p* = 0.033) and MIF 16 weeks after initiation of anti-TNF antibody treatment (*r* = 0.31, *p* = 0.049) (Figure 2 D and E, respectively).

### Chemerin Serum Levels at Baseline are not Related to Traditional Risk Factors of Atherosclerosis

**Figure 3:**
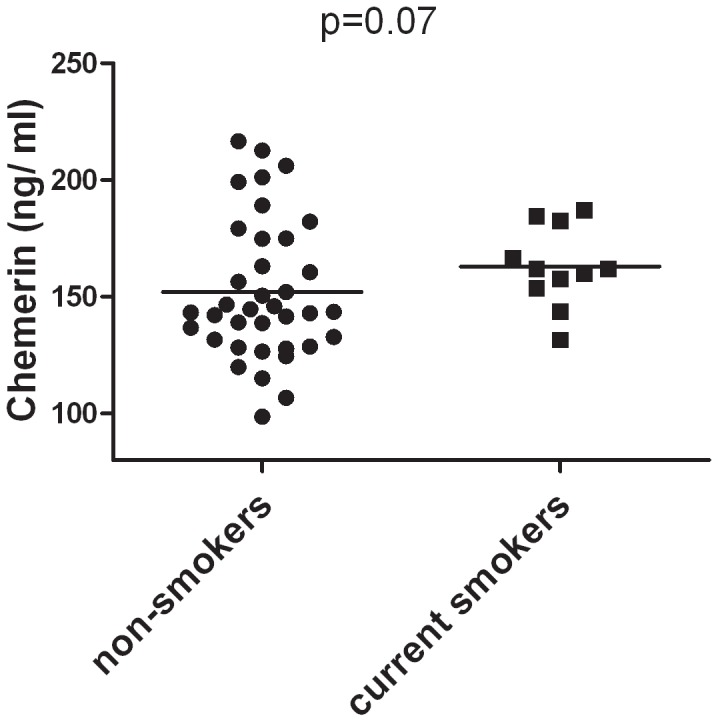
Chemerin levels at baseline tend to be higher in current smokers (n = 11) than in non-smokers (n = 38). Data are presented as median values.

Chemerin serum levels at baseline were not related to sex, age, BMI, lipid profile (TC, HDL, LDL, TG, Apo A-I, Apo B, Lp(a)), systolic or diastolic blood pressure, cardiovascular events in the medical history, diabetes or the use of statins and anti-hypertensive drugs, or was the change in chemerin levels 16 weeks after initiation of adalimumab treatment related to the change in lipid profile. Of interest, we found a trend towards higher levels of serum chemerin in current smokers (n = 11, 162 ng/ml, IQR154–183 ng/ml) than in non-smokers (n = 38, 143 ng/ml, IQR 129–175) (*p* = 0.07) (Figure 3). In this cohort smoking was not associated with increased disease activity or a diminished response to therapy.

## Discussion

In this exploratory study chemerin serum levels decreased significantly after initiation of adalimumab treatment and this decrease was modestly associated with the decrease in disease activity parameters DAS28, ESR and CRP and with the inflammatory mediators IL-6 and MIF. Of importance, chemerin serum levels only showed a statistically significant decrease in EULAR responders. In accordance with our findings, previous studies reported positive associations of chemerin serum levels and markers of inflammation [16], [17] and correlations between the decrease in chemerin serum levels and the decrease in CRP levels. [18] Furthermore, the reduction in chemerin levels was modestly associated with the reduction in IL-6 serum levels and MIF serum levels. Of importance, in RA macrophages are a source of IL-6 and MIF and the decrease in the number of synovial macrophages is consistently associated with clinical improvement after initiation of anti-TNF therapy. [19] As chemerin is a potent chemoattractant for macrophages, TNF antagonists might partly exert their effect by reducing chemerin levels, thereby reducing the number of synovial macrophages. In addition to its chemotactic function it was recently shown that chemerin can induce the release of IL-6 by RA FLS. [7] Thus, the decreased IL-6 serum levels observed after TNF blockade might be partly explained by decreased chemerin levels resulting in decreased FLS-derived IL-6. On the other hand, as TNF upregulates chemerin production by RA FLS [7], by blocking TNF, this cytokine will not be able to induce chemerin production by FLS, resulting in decreased chemerin serum levels.

In contrast to some previous studies [16], [17], we did not find a clear cut relationship between traditional risk factors for atherosclerosis and chemerin serum levels, suggesting that chemerin might represent an independent risk factor. Interestingly, the prevalence of traditional risk factors does not account for the increased risk of cardiovascular morbidity and mortality in RA patients and chronic inflammation, characterizing RA, is considered to play a key role in accelerating atherosclerosis. [20].

We also found a modest association between the decrease in chemerin serum levels and the decrease in MIF serum levels 16 weeks after initiation of TNF blockade. Interestingly, MIF has recently emerged as an important cytokine possibly linking RA and atherogenesis [13], [21]. It is tempting to speculate that the decrease of chemerin levels after TNF blockade will result in the decreased of MIF by inhibiting the recruitment of MIF-producing cells (i.e. macrophages and DC), contributing to reduced vascular inflammation.

Consistent with data obtained in a murine model for cigarette smoking [22], our data suggest that smoking might elevate chemerin serum levels. Furthermore, in ChemR23 knockout mice, exposure to cigarette smoke resulted in diminished recruitment of inflammatory cells (neutrophils, inflammatory monocytes, DC and T cells) and decreased levels of chemokines (CCL2, CXCL1 and CCL20) in bronchoalveolar lavage fluid and lung tissues compared to those from wild-type mice, suggesting involvement of the chemerin/ChemR23 axis in inflammation secondary to to smoking. In RA cigarette smoking is a well-recognized risk factor for the development of the disease and it has also been associated with more severe disease. [23] Thus, elevated expression of chemerin might represent one of the underlying mechanisms by which smoking increases disease activity in RA patients.

An obvious limitation to this study is the relatively small number of patients, allowing only univariate analysis. However, it is important to mention that treatment and response evaluation were conducted according to a stringent treatment protocol.

In conclusion, we have shown that adalimumab treatment reduced chemerin serum levels which are associated with the reduction in disease activity parameters (DAS28, ESR and CRP), and the inflammatory mediators IL-6 and MIF. As these mediators are produced by macrophages, their reduction might be a consequence of decreased chemerin-dependent accumulation of synovial macrophages. This suggests a distinct role for chemerin in the migration and/or retention of macrophages in the synovium and as such in the pathophysiology of RA. Altogether, the present study suggests that the sustained downregulation of chemerin is associated with mechanisms by which anti-TNF therapy might reduce (vascular) inflammation in RA patients.

## Supporting Information

Checklist S1 **CONSORT 2010 Checklist.**
(DOC)

Enrollment log S1 **Trial enrollment log including reason of drop out/harms.**
(XLS)

Protocol S1 Trial protocol: “Prospective study on the effects of adalimumab treatment in patients with rheumatoid arthritis who are naïve for TNF-α blocking therapy and patients who do not respond (anymore) to prior treatment with other anti-TNF-α medication.”
(DOC)
