# Supplementary material for: Anti-TNF Therapy Reduces Serum Levels of Chemerin in Rheumatoid Arthritis: A New Mechanism by Which Anti-TNF Might Reduce Inflammation
Source: PLoS One. 2013 Feb 27;8(2):e57802. doi: 10.1371/journal.pone.0057802 (PMC3584053; doi:10.1371/journal.pone.0057802)
Supplement: Protocol S1 — Trial protocol: “Prospective study on the effects of adalimumab treatment in patients with rheumatoid arthritis who are naïve for TNF-α blocking therapy and patients who do not respond (anymore) to prior treatment with other anti-TNF-α medication.” (DOC) [file pone.0057802.s003.doc]

Prospective study on the effects of adalimumab treatment in patients with rheumatoid arthritis who are naïve for TNF-α blocking therapy and patients who do not respond (anymore) to prior treatment with other anti-TNF-α medication.

Protocol version 2.1

04-02--2008

Carla Wijbrandts, MD

Dorine Sijpkens, MSc

Daniëlle Gerlag, MD

Paul P. Tak, MD, PhD

Division of Clinical Immunology and Rheumatology, F4-218

Department of Medicine,

Academic Medical Center/University of Amsterdam

Meibergdreef 9, 1105 AZ Amsterdam, The Netherlands

Fax: +31 20 691 9658 Tel: +31 20 566 2171 E-mail:P.P.Tak@amc.uva.nl

| *Schedule of Observations* |  | Screening | T=-2 | T=0 | T=4 | T=16 | T=28 | T=40 | T=52 |
| --- | --- | --- | --- | --- | --- | --- | --- | --- | --- |
| *Visit number* |  | **V0** | **V1** | **V2** | **V3** | V4 | **V5** | **V6** | **V7** |
| TNO/insurance request for approval | *(DAS 28)* | X |  |  |  | X |  |  |  |
| Demographics | *Age*  *Race*  *Height*  *Weight*  *Alcohol*  *Smoking* |  | X |  |  |  |  |  |  |
|  |  |  |  |  |  |  |  |  |  |
| DMARD & biological history | *DMARDs*  *Prednison*  *Biologicals* |  | X |  |  |  |  |  |  |
| Concommitant. medication |  | X | X | X | X | X | X | X | X |
| In- and exclusioncriteria |  | X | X |  |  |  |  |  |  |
| Informed consent |  |  | X |  |  |  |  |  |  |
| mRNA for storage |  |  |  | X | X | X | X | X | X |
| DNA sample (genetic polymorphisms) |  |  |  | X |  |  |  |  |  |
| X-thorax (< 3 mnd voor baseline) |  |  | X |  |  |  |  |  |  |
| PPD (<6 mnd voor baseline) |  |  | X |  |  |  |  |  |  |
| Virology screening | *Hepatitis B, C, HIV* |  | X |  |  |  |  |  |  |
| Urine β-HCG | *pregnancy test* |  | X |  |  |  |  |  |  |
| IgM RF, anti- CCP, ANA, ds-DNA |  |  | X |  |  |  | X |  | X |
| Radiological progression | *Sharp modified by*  *Van der Heijde score* |  |  | X (<3mnd accepted) |  |  |  |  | X |
| (X-hands, feet) |  |  |  |  |  |  |  |  |  |
| Routine laboratory testing | *ESR, CRP, Hb, Ht, Leuco’s, Trombo’s, creatinin, ureum, Na, K, ALAT (SBPT), ASAT (SGOT), LDH, γGt, AF, glucose, bilirubine, alkaline phosphatase, protein and albumin,* |  | X | X | X | X | X | X | X |
|  | *Urine dipstick: glucose, protein, blood* |  |  |  |  |  |  |  |  |

| *Schedule of Observations* |  | Screening | T=-2 | T=0 | T=4 | T=16 | T=28 | T=40 | T=52 |
| --- | --- | --- | --- | --- | --- | --- | --- | --- | --- |
| *Visit number* |  | **V0** | **V1** | **V2** | **V3** | **V4** | **V5** | **V6** | **V7** |
| Disease activity | *DAS 28*  *ACR*  *RADAI*  *HAQ-score*  *VAS pain*  *VAS dis.act*  *Dis act Phys/observer*  *Dis act Pat*  *Morning stiffness*  *Short form health survey (SF 36)* |  |  | X | X | X | X | X | X |
| Bonemineraldensity | *DEXA-scan* |  |  | X |  |  |  |  | X |
| Serum for storage | *serologic markers, anti-antibodies, markers of boneformation & resorption* |  | X | X | X | X | X | X | X |
| Urine for storage | *markers for boneresorption & cartilage degradation* |  | X | X | X | X | X | X | X |
| Vital signs | *BP, pulse, temperature (oraal), weight, height* |  | X | X | X | X | X | X | X |
| Employement status | *jobstatus, sickleave, work resumation, % work* |  |  | X |  | X |  |  | X |
| Safety checks | *Infection , malignancy, hospital admittance, adverse events* |  |  | X | X | X | X | X | X |

Table of contents

[1. Introduction. 4](#__RefHeading___Toc60568766)

[Targeted therapy by TNF-α blockade in RA. 4](#__RefHeading___Toc60568767)

[Objectives 6](#__RefHeading___Toc60568768)

[Research question(s) 6](#__RefHeading___Toc60568769)

[2. Investigational Plan 7](#__RefHeading___Toc60568770)

[Study medication and dosage 7](#__RefHeading___Toc60568771)

[Design. 7](#__RefHeading___Toc60568772)

[Duration of study 8](#__RefHeading___Toc60568773)

[Inclusion criteria: 8](#__RefHeading___Toc60568774)

[Exclusion criteria: 9](#__RefHeading___Toc60568775)

[Screenings procedures 10](#__RefHeading___Toc60568776)

[Variables and schedule of observations 10](#__RefHeading___Toc60568777)

[Background information 10](#__RefHeading___Toc60568778)

[Primary endpoint 11](#__RefHeading___Toc60568779)

[Secondary endpoints 11](#__RefHeading___Toc60568780)

[**Safety Variables** 12](#__RefHeading___Toc60568781)

[Adverse events 12](#__RefHeading___Toc60568782)

[**Laboratory variables** 12](#__RefHeading___Toc60568783)

[**Additional variables** 13](#__RefHeading___Toc60568784)

[**Clinical variables** 13](#__RefHeading___Toc60568785)

[Concomitant illnesses 14](#__RefHeading___Toc60568786)

[Concomitant treatment 14](#__RefHeading___Toc60568787)

[Concomitant medications 15](#__RefHeading___Toc60568788)

[Infections 16](#__RefHeading___Toc60568789)

[Pregnancy 16](#__RefHeading___Toc60568790)

[Overdosage 16](#__RefHeading___Toc60568791)

[Adverse events 17](#__RefHeading___Toc60568792)

[*Definition of serious and non-serious adverse events* 18](#__RefHeading___Toc60568793)

[*Reporting/documentation of adverse events* 18](#__RefHeading___Toc60568794)

[*Assessment of severity* 19](#__RefHeading___Toc60568795)

[Withdrawl and replacement of patients 19](#__RefHeading___Toc60568796)

[Premature discontinuation of study 19](#__RefHeading___Toc60568797)

[3. Methods of investigation 20](#__RefHeading___Toc60568798)

[Clinical examinations 20](#__RefHeading___Toc60568799)

[Genetic polymorphisms 20](#__RefHeading___Toc60568800)

[Bonemetabolism 20](#__RefHeading___Toc60568801)

[Bonemineraldensity 21](#__RefHeading___Toc60568802)

[Radiologic progression 21](#__RefHeading___Toc60568803)

[Disease activity 21](#__RefHeading___Toc60568804)

[Employment 21](#__RefHeading___Toc60568805)

[Data analysis 21](#__RefHeading___Toc60568806)

[4. Ethical and legal aspects 22](#__RefHeading___Toc60568807)

[Good clinical practice 22](#__RefHeading___Toc60568808)

[Informed consent of patient 22](#__RefHeading___Toc60568809)

[Approval of study protocol 22](#__RefHeading___Toc60568810)

[Confidentiality 22](#__RefHeading___Toc60568811)

[Liability and insurance 23](#__RefHeading___Toc60568812)

[Use of study findings 24](#__RefHeading___Toc60568813)

[5. Protocol amendments 24](#__RefHeading___Toc60568814)

[6. Appendix I Classification of Functional Status 27](#__RefHeading___Toc60568815)

[7. Appendix II Declaration of Helsinki 28](#__RefHeading___Toc60568816)

# 1. Introduction.

Although the etiology of rheumatoid arthritis (RA) remains elusive, immune-mediated mechanisms are likely of crucial importance. The evidence to support a role of CD4+ T-cells in the immune response in RA patients is substantial, although circumstantial. It has become clear that cells other than lymphocytes, in particular activated macrophages, play a critical role as effector cells in chronic disease (1). These cells are highly activated and secrete a variety of cytokines as well as matrix metalloproteinases. The macrophages often constitute the majority of the inflammatory cells in the synovial sublining (2). The importance of these cells and their soluble mediators is supported by clinical observations. Local disease activity is particularly associated with the number of macrophages and the expression of cytokines, such as TNF-α and IL-6, in synovial tissue (3). There is also a significant positive correlation between intimal lining layer depth and cell counts for macrophages in the synovial sublining on the one hand and radiographic signs of joint destruction after follow-up on the other (4). The pivotal role of TNF-α - at least in the majority of RA patients - has been confirmed by the impressive effects of specific therapeutic strategies targeting the TNF-α molecule (5-9).

## Targeted therapy by TNF-α blockade in RA.

Therapy with TNF-α blockade plus methotrexate may provide sustained clinical benefit in patients with active RA despite previous therapy with methotrexate. This combined therapy may not only control the symptoms and signs of RA effectively, but may also improve the quality of life (7). Therapy with the chimeric monoclonal antibody infliximab plus methotrexate prevents progressive joint damage and results in improvement in radiographic scores of joint damage in a significant percentage of patients. The combination of infliximab and methotrexate could halt the progression of joint damage not only in patients with limited joint damage, but also in those with extensive damage, whereas conventional therapies only have a moderate effect on progression of the disease. Still, at least 30% of the patients do not respond to infliximab therapy with regard to arthritis activity (5;7). One of the explanations could be that patients develop anti-infliximab antibodies (human anti-chimeric antibodies, HACA’s) after initiation of treatment. Consistent with this notion we have recently shown that the formation of such antibodies may lead to infusion related allergic reactions (10) and lack of clinical response (14). The inhibitory antibodies are of IgG1 and IgG4 subclasses (unpublished data). The observed class switched B cell response points to a T cell dependent humoral response. Therefore there can be specific T helper epitopes in the adalimumab sequence against which T cel reactivity may be induced.

This can give insight in the T cell response involved in antibody formation against the anti-TNFα therapeutic antibodies. This will allow either modification of the therapeutic antibodies to prevent inhibitory antibody formation or patient monitoring at the start and during treatment.

Adalimumab is a novel recombinant human IgG1 monoclonal antibody specific for human TNF-α. Adalimumab was created using phage display technology resulting in an antibody with human derived heavy and light chain variable regions and human IgG1:К constant regions. Adalimumab is produced by recombinant DNA technology in a mammalian cell expression system and is purified by a process that includes specific viral inactivation and removal steps. It consists of 1330 amino acids and

has a molecular weight of approximately 148 kilodaltons. Adalimumab has been approved by the FDA and EMEA and is now available in the Netherlands *(for detailed information see appendix).*

We have treated RA patients with adalimumab as part of our routine clinical practice; these patients did not fulfill the Eular response criteria (DAS 28) for clinical improvement after infliximab treatment. Of importance, the majority of these patients do respond to adalimumab treatment. Conceivably, some of these patients have developed human anti-chimeric antibodies, which affect infliximab treatment, but not adalimumab treatment.

The goal of this study is to follow up patients who will initiate adalimumab as their first TNF-α blocking biological treatment and patients who initiate adalimumab after they failed other TNF-α blockers. We will look for clinical parameters and serological markers that may differentiate responders from non-responders on adalimumab. We will evaluate changes in disease activity in relationship to adalimumab treatment by monitoring both clinical signs of arthritis activity and markers of bone and cartilage destruction. Furthermore we will measure antibodies formed against prior TNF-α blockers before initiating adalimumab in patients who failed prior anti-TNF-α treatment. This will allow us to determine the role of anti-antibody formation in the response to TNF-α blockade. In addition, in an exploratory setting we will collect biological data to study the effects of factors other than anti-antibody formation that could affect the clinical response. Conceivably, a subset of patients has disease primarily driven by cytokines other than TNF-α. The minority of patients who fail both the prior TNF-α blocker, and adalimumab might fit in this category and we will try to identify the driving immunological mechanisms in these patients.

## Objectives

To evaluate the response to adalimumab treatment in TNF-α blockade naïve patients and patients who failed prior other anti-TNF-α treatment and to understand the mechanisms underlying the clinical response to TNF-α blockade.

Research question(s)

1. *What factors distinguish responding patients from non responding patients on adalimumab treatment? (anti-(anti-TNF-*α*) antibody formation, differences in cytokine profiles or other serological markers, RA activity driven by other mediators than TNF-*α*)*

2) What percentage of patients (TNF-α blockade naïve versus failures on prior anti-TNF-α treatment) respond after 16 weeks of adalimumab treatment?

*3) What is the clinical efficacy of adalimumab after 1 year treatment?*

*(Eular response criteria (DAS 28), ACR response, RADAI, SF 36, HAQ, and radiological progession)*

*4) Can we find genetic markers, e.g. genetic polymorphisms in the TNF-*α *genes, that may predict diagnosis, efficacy and side-effects of treatment in the individual patient?*

*5) Can periferal blood (mRNA) micro-array analysis identify new markers that distinguish responders from non-responders to adalimumab treatment?*

*6) What are the effects of adalimumab on bonemineraldensity (DEXA) and bone metabolism (serum and urine markers for boneformation and resorption)?*

*7) What are the effects of adalimumab on functioning at work and sickleave?*

*8)* *Are inhibitory antibodies formed in a T cell dependent humoral response and if so, which are the specific epitopes in the adalimumab sequence recognized by T cells involved in antibody formation against itself?*

#

# 2. Investigational Plan

## Study medication and dosage

**Generic name: adalimumab**

**Trade name: Humira®**

Dosage: 40 mg (0,8 ml) by subcutaneous injection at baseline and every other week.

After succesfull training of the injection technique at the out-patient clinic under supervision of a special trained nurse or physician, patients are allowed to administer adalimumab at home.

There will be a ± 7-day deviation for all return visits.

All visits will be fixed with reference to the baseline visit.

The study medication will be on prescription (according to the guidelines of “de Nederlandse Vereniging voor Reumatologie” and the manufacturer’s instructions).

## Design.

A monocenter prospective, exploratory study with a 1 to 2-week screening period and a 52-week follow-up period in 120 RA patients who receive adalimumab treatment in routine rheumatological practice and are TNF-α blockade naïve or have failed to (or no longer) respond to other anti-TNF-α treatment. There is no group of patients receiving control treatment, since it is considered unethical to withhold active treatment for 52 weeks in patients with active RA. Previous work has shown the efficacy of adalimumab in randomized trials (8;9).

The goal of the present study is to evaluate the response to adalimumab treatment in the TNF-α blockade naïve patients and patients who failed on other prior TNF-α blocking treatment. Therefore, responders will be compared with non-responders after all included patients have received anti-TNF-α therapy in the form of adalimumab. Furthermore the goal is to understand more of the mechanisms underlying the response to anti-TNF-α treatment. Adalimumab treatment will be discontinued in patients who do not fulfill the Eular response criteria for improvement (DAS 28) at week 16, according to routine clinical practice.

Patients who discontinue adalimumab at or after week 16 for lack of respons or other reasons will get a final clinical assessment as part of the close out visit. The reason for discontinuation will be noted in the case record form. These patients will not be followed for measurement of clinical parameters. In patients who respond, treatment will be continued and may be extended after the 52-week follow-up period of this study. Patients will be included starting January 2004.

Patients who drop out of the study before week 16 for any reason will be replaced by new patients, the reason of discontiuation will be noted in the case record form (*see withdrawl and replacement of patients)*

## Duration of study

All patients participating in this study will receive 40 mg (0,8ml) adalimumab by subcutaneous injection every other week after fulfilling all inclusion criteria at baseline. All patients must have approval for reimbursement for TNF-blockade by their insurance company (DAS 28 >3.2). Patients will be seen during the study for efficacy and safety assessments at weeks 4, 16, 28, 40 and 52 using validated and generally accepted evaluation criteria (see laboratory and clinical variables). All visits will be fixed with reference to the baseline visit. Evaluation of ACR 20 and Eular response (DAS 28) will be performed at 16 weeks after initiation of adalimumab. The final clinical assessments will be performed at 52 weeks. If the patient does not meet the Eular criteria for moderate or good response at 16 weeks, adalimumab treatment is discontinued and no further assessments will be done.

Inclusion criteria:

1) Patients with the diagnosis rheumatoid arthritis according to the American Rheumatism Association (ARA) 1987 criteria and in ACR 1991 functional classes I, II, and III *(see appendix)*

2) The patient is naïve for anti-TNF-α therapy or has failed other prior TNF-α blockers

3) DAS 28 ≥ 3.2

4) Age > 18 and ≤ 85 years old

5) Use concurrent methotrexate treatment (5 - 30 mg/week; stable since at least 28

days before initiation) during the study. Subjects may be taking nonsteroidal anti-

inflammatory drugs, provided the dose and frequency have been stable for at

least 28 days. Subjects may be receiving prednisone therapy ≤ 10 mg/day

provided that the dosage has been stable for at least 2 months prior to entry.

### Exclusion criteria:

1. Pregnancy
2. Breastfeeding
3. A history of or current acute inflammatory joint disease of different origin e.g. mixed connective tissue disease, seronegative spondylarthropathy, psoriatic arthritis, Reiter’s syndrome, systemic lupus erythematosus or any arthritis with onset prior to age 16 years
4. Acute major trauma

5) Therapy within the *previous 60 days* with:

- any experimental drug
- alkylating agents, e.g. cyclophosphamide, chlorambucil
- antimetabolites
- monoclonal antibodies (including infliximab and etanercept)
- growth factors
- other cytokines

6) Therapy within the *previous 28 days* with:

- parenteral or intraarticular corticoid injections
- oral corticosteroid therapy exceeding a prednisone equivalent of 10 mg daily
- present use of DMARDs other than methotrexate

7) Receipt of any live (attenuated) vaccines within 4 weeks prior to baseline

8) Fever (orally measured > 38 °C), chronic infections or infections requiring anti-

microbial therapy

9) Known positive reaction to hepatitis B surface antigen or Hepatitis C antigen

10) Other active medical conditions such as inflammatory bowel disease, bleeding

diathesis, or severe unstable diabetes mellitus

11) Manifest cardiac failure (stage III or IV according to NYHA classification)

12) Progressive fatal disease/terminal illness

13) A congenital or acquired (known HIV-positive status) immunodeficiency

1. 14) a history of lymphoproliferative disease or treatment with total lymphoid

irradiation.

15) A white cell count less than 3.5 x 10^9^/l

16) Platelet count less than 100 x 10^9^/l

17) Haemoglobin of less than 5.3 mmol/l

18) Body weight of less than 45 kg

19) History of drug or alcohol abuse

20) Any concomitant medical condition which would in the investigator’s opinion compromise the patient’s ability to tolerate, absorb, metabolize or excrete the study medication.

21) Inability to give informed consent

22) Mental condition rendering the patient unable to understand the na­ture, scope

and possible consequences of the study and/or evidence of an uncooperative attitude.

Screenings procedures

Screening will be done within 1-2 weeks before baseline to assess the in- and exclusion criteria and to evaluate if all the safety parameters are met. All patients sign their informed consent form before baseline.

Variables and schedule of observations

The following will be determined in accordance with the "Schedule of observations" *(see page 2-3*).

### Background information

Before enrolment in the study, all patients will undergo a full physi­cal examination, and the relevant medical history will be taken. Further to this demographic data will be collected/measured and documented.

The following data will be documented:

- date of obtaining informed consent
- initials, date of birth, sex, race, weight and height
- alcohol consumption and smoking behaviour
- concomitant illnesses, infections, malignancies and treatment
- rheumatoid arthritis disease status and time since RA diagnosis
- DMARD history including infliximab and etanercept, current medication
- changes in dosage of prednison, NSAIDs and methotrexate
- relevant findings in physical examination

### Primary endpoint

The primary endpoint of the study is the percentage of patients with a moderate to good response to adalimumab treatment at 16 weeks according to the Eular response criteria which is also applied in routine rheumatological practice.

A decrease in the DAS 28 value must be ≥ 1.2 at any given timepoint after initiation of treatment for continuation of reimbursement by the insurance company.

| **DAS 28 score at**  **(week 16)** | **Improvement in DAS 28 at week 16 compared to baseline (week 0)** | | |
| --- | --- | --- | --- |
|  | **>1.2** | **> 0.6 en ≤ 1.2** | **≤ 0.6** |
| **≤ 3.2** | **Good** |  |  |
| **> 3.2**  **and ≤ 5.1** |  | **Moderate** |  |
| **> 5.1** |  |  | **None** |

*Tabel 1: Eular response criteria*

Furthermore the primary endpoint of the study is to search for clinical parameters and/or serological markers that possibly distinguish responders from non-responders to TNF-α blockade by adalimumab.

### Secondary endpoints

The secundary endpoint of the study is the proportion of patients with a 20%, 50%, and 70% clinical improvement according to the ACR response criteria (11) at 4, 16, 28, 40 and 52 weeks after the initiation of adalimumab treatment. The parameters of disease activity e.g. tender joint count, swollen joint count, patient’s assessment of pain, patient’s global assessment, investigators global assessment, duration of morning stiffness, health assessment questionnaire, DAS 28, SF-36 (short form health survey) and RADAI (12) will be assessed at each time point starting from baseline. Also an assessment of the influence of adalimumab treatment on physical and mental wellbeing in relation to employment status, job-satisfaction and sickleave will be implemented in the form of questionnaires.

In addition we will look at the influence of adalimumab on bonemineraldensity, bonemetabolism.

This will be done by analysing serum and urine markers for bone formation and resorption, performing a DEXA scan before- and one year after adalimumab initiation.

We will look for genetic markers, e.g. genetic polymorphisms in TNF-α genes, that may predict diagnosis, efficacy and side-effects of treatment in the individual patient.

In the future micro-array analysis may be done to screen for new markers that distinguish responders from non-responders. Also, immunological responses raised against adalimumab antibodies will be researched, comprising antibody formation and T cell responses.

## **Safety Variables**

### Adverse events

Adverse events observed by the investigator or reported by the patient will be documented as described below in this study protocol. Infections, malignancies and hospitalisation during adalimumab will be noted on a separate page in the case record form.

## **Laboratory variables**

The following laboratory variables will be assessed prior to baseline.

HEMATOLOGY: hemoglobin, haematocrit, red blood cell count, white blood cell count (and if abnormal, differential count), platelet count.

chemistry: glucose, creatinine, sodium, potassium, calcium, phosphate, uric acid, bilirubin (total), alkaline phosphatase, ASAT(SGOT), ALAT(SGPT), LDH, γGT, CPK, total protein and albumin, CRP.

urinalysis: semiquantitative (dipstick) for glucose, protein, blood. If protein is positive urine sediment should be done and urinary protein loss (over 24 hours) should be determined quantitatively. Pregnancy test in women with child-bearing potential (β-HCG).

IMMUNOLOGY: ANA, IgM RF, anti ds-DNA, and anti-CCP

at week 0 and week 28 and 52 (also performed at close out visit)

VIROLOGY: HbsAg, HCV and HIV-test at pre-screening

## **Additional variables**

Chest-X ray (<3 months old is acceptable)

PPD skin test (repeat if prior PPD is 6> months ago)

If the PPD skin test is positive, but there are no signs of an active TB on the chest X-ray and the patient does not have any symptoms of an active TB, the patient is allowed to enter the study, but must be treated for possible latent TB prior to the first dose of adalimumab in the trial.

If the PPD skin test is positive and signs of an active TB are visible on the chest X-ray or the patient has symptoms of an active TB, the patient is not allowed to enter the study.

In this study per patient a total volume of approximately 215 ml of blood will be drawn for study purposes over a period of 52 weeks.

## **Clinical variables**

As clinical safety monitoring the following examinations will be performed:

A complete medical history will be taken before enrollment. Interest will be focused on current concomitant diseases and DMARD-history.

Physical examination will be performed at screening. Vital signs (blood pressure, pulse frequency, body weight, oral body temperature) will be determined at each visit.

Any evaluations that are abnormal and considered to be related to the study medication will be followed until they return to baseline or are judged not to be clinically significant.

### Concomitant illnesses

Additional illnesses present at entry to the study are regarded as con­comitant illnesses and will be documented on the case record form pro­vided.

Any concomitant disorder such as diabetes, hypertension, heart failure etc. should be controlled before entry into the study by appropriate medication which should, if possible, not be changed during the entire study period.

Patients with congestive heart failure stadium III of IV according to the NYHA classification will be excluded from the study.

Concomitant disorders, current medication and any changes in concomitant disorders and/or methotrexate, NSAID or prednison dosage have to be documented appropriately in the case record forms.

Illnesses occurring during the study period are to be regarded as adverse events and will each be documented in the medical records. Adverse events will be documented on a separate "Adverse event" page in the case record form. Especially infections, malignancies and hospitalisation will be noted on a separate page in the case record form.

Application of study medication may be continued during intercurrent illnesses - if clinically justifiable and as long as none of the withdrawal criteria is met.

### Concomitant treatment

All treatments being taken by the patients on entry to the study and all treatments given in addition to the study treatment during the study are regarded as concomitant treatments and must be docu­­mented on the case record form.

### Concomitant medications

Should be kept to a minimum during the study. However, if these are considered necessary for the pa­tient's welfare and will not interfere with the study medication, they may be given at the discretion of the investigator. If the patient is responding to adalimumab treatment, tapering of the prednisone is allowed after week 16.

The following concomitant treatments (for RA) are permitted during this study, provided that the patients have received a stable dose for at least 28 days before entering the study:

- nonsteroidal anti-inflammatory drugs (NSAIDs) including acetylsalicylic acid
- oral steroids, if not exceeding a prednisone equivalent of 10 mg daily (stable for 2 months prior to entry)
- methotrexate (5-30 mg/week, obligated in all patients)

The following concomitant treatments are not permitted during this study (see exclusion criteria):

- intra-articular corticosteroid injections
- oral steroids, if exceeding a prednisone equivalent of 10 mg daily
- DMARDs other than methotrexate
- any alkylating agents (e.g. cyclophosphamide, chlorambucil)
- any anti-metabolites
- any other monoclonal antibodies, growth factors, or cytokines
- any experimental drug

It is important that steroid treatment be kept to a minimum. Changes of NSAIDs and methotrexate should be avoided. Decreases in steroid therapy (drug or dosing) are allowed after evaluation of response at 16 weeks. If there is a clear reason to do so, the reason and the changes have to be documented in the case record form.

If the administration, a change in dosing or the stopping of any concomitant treatment is necessary, it must be reported in the patient's medical records and the case record form. As far as possible, no changes should be made to the concomitant treatment during the study.

Patients undergoing surgical procedures involving general anesthesia may interrupt study medication. In total, a single dosing interruption up to 10 days is allowed (see also *Withdrawl and replacement of patients*).

Patients requiring surgical procedures involving local or regional anesthesia may continue with study medication.

Infections

Serious and opportunistic infections have been described in patients receiving TNF blocking agents, but it is not clear that their incidence is higher than in patients with rheumatoid arthritis using other forms of DMARD therapy and/or corticosteroids. TNF blocking agents should not be started or should be discontinued when serious infections occur, including septic arthritis, infected prostheses, acute abscess, osteomyelitis, sepsis, systemic fungal infections, Listeria etc. Treatment with TNF-blockers in such patients should only be resumed if the infections have been treated adequately.

Pregnancy

An embryo-fetal perinatal developmental toxicity study has been performed in monkeys and has revealed no evidence of harm to the fetuses due to adalimumab, however no adequate and well-controlled studies in pregnant women have been performed. For this reason pregnancy is an exclusion criterium in this study.

Overdosage

The maximum tolerated dosage of adalimumab has not been established in humans. Multiple doses up to 10mg/kg have been administered to patients in clinical trials without evidense of dose-limiting toxicities. In case of an overdosage, the patient will be monitored for any signs or symptoms of adverse reactions or effects and treated when necessary.

Adverse events

An adverse event is any event which impairs the wellbeing of the patient during the period of observation in a clinical study, including intercur­rent illness or accident. The term ad­verse event does not imply a causal re­lation­ship with the study treat­ment.

The period of observation for adverse events extends from the time the patient gives informed consent until he or she undergoes a final exami­nation as part of the study, including periods before and after an active treatment phase during which no drug or other treatment is given. The period may also include a specified number of days after the final ex­amination during which time serious adverse events must be re­ported (see below). Adverse events occurring after the end of the study must be re­ported if the investigator considers there is a causal relationship with the study medica­tion.

All patients experiencing adverse events ‑ whether considered as­soci­ated[[1]](#footnote-2) with the use of the study medication or not ‑ must be monitored until symptoms subside and any abnormal laboratory values have re­turned to baseline, or until there is a satis­factory explanation for the changes ob­served, or until death, in which case a full pathologist's re­port should be supplied, if possible. All findings must be reported in the patient's medi­cal re­cords.

All drug-related adverse events, including intercurrent illnesses, must be reported on and docu­mented as described below.

Adverse events are divided into the categories "serious" and "non­seri­ous". This determines the procedure that must be used to re­port/document the adverse event (see below).

*Definition of serious and non-serious adverse events*

A serious adverse event is any adverse event occurring at any dose that results in:

1. Death
2. A life-threatening event
3. Inpatient hospitalization or prolongation of existing hospitalization
4. Persistent or significant disability/incapacity
5. Congenital anomaly/birth defect

Adverse events that do not fall into these categories are defined as non-serious.

*Reporting/documentation of adverse events*

*Serious adverse events*

All serious adverse events which occur during the period of observation, whether consid­ered to be associated with the study medication or not must also be docu­mented in the case record form.

Where necessary, inves­tigators will in­form the medical ethics committee and authorities.

*Non-serious adverse events*

These are to be documented in the case re­cord form if related with the study medication.

*Adverse events occurring after the period of observation*

Any adverse event occurring at any time after the end of the study and considered to be caused by the study medication - and therefore a possible adverse drug reac­tion - must be re­ported as above.

*Assessment of severity*

Regardless of the classification of an adverse event as serious or non-se­ri­ous (see above), its severity must be assessed as mild, moderate or se­vere, according to medical criteria alone:

mild = does not interfere with routine activities

moderate = interferes with routine activities

severe = impossible to perform routine activities

It should be noted that a severe adverse event need not be serious in nature and that a serious adverse event need not, by definition, be se­vere. Regardless of se­verity, all serious adverse events must be re­ported as above.

### Withdrawl and replacement of patients

Patients who drop out of the study before week 16 for any reason will be replaced by new patients, the reason of discontiuation will be noted in the case record form.

These patients will be seen for a final clinical assessment as part of the close out visit.

This visit will include physical examination, final assessment of disease activity (ACR, DAS 28, RADAI, SF 36), follow up of previous adverse events. Blood will be drawn for safety screening of clinical heamatology and chemistry parameters. Furthermore final immuno- serology to screen for auto-antibody formation (ANA, anti-ds DNA). Titers of IgM-RF and anti-CCP will also be drawn at this visit.

Premature discontinuation of study

The investigator has the right to discontinue this study at any time for medical and/or administrative reasons. As far as possible, this should occur after mutual consultation. Furthermore adalimumab treatment will be discontinued if there is no reimbursement by the insurance company.

# 3. Methods of investigation

Clinical examinations

The clinical examinations including vital signs as well as safety assessments will be performed as usual in routine clinical practice. Blood will be drawn at the 7 visits 1 EDTA tube, 1 lithium heparine tube, 2 serum tubes and a 2.5 ml tube mRNA for storage at each time point. Serum will be immediately frozen at -70°C. The material will be used for detection of acute phase reactants, biologic markers (such as cytokines), anti-antibodies, leukocyte differential, and gene activation (micro array) in relation to the disease.

All samples must be clearly *labeled* (using labeling that can withstand freezing in liquid nitrogen/dry ice) including patient number and date and time of sampling.

Routine bloodtests for haematology and chemistry will be performed each visit including ESR and CRP as measures of disease activity. Routine urine samples are taken to check for proteinuria, blood and leucocyten and dipstick testing.

At baseline, after 28 and 52 weeks immunological assays e.g. anti-ds DNA (Crithidia), IgM rheumatoid factor, ANA and anti-CCP will be performed. This will be 1 serum tube at 3 visits.

Genetic polymorphisms

Only at baseline 1 additional EDTA tube will be drawn for DNA isolation and study of genetic markers, e.g. genetic polymorphisms in the TNF- alpha genes that may pedict efficacy and side-effects of treatment.

Bonemetabolism

Urine samples will be frozen from 6 visits for analysis of markers involved in bone resorption e.g. CTX I and CTX II for cartilage degradation.

Other markers for bone formation e.g. osteocalcin will be determined in the serum.

Bonemineraldensity

A DEXA scan will be performed at baseline and at 52 weeks to compare bonemineraldensity changes after adalimumab treatment.

Radiologic progression

X-rays of hands and feet will be performed at baseline and 52 weeks to evaluate disease progression using the modified Sharp v.d. Heijde scoring method.

Disease activity

This will be assessed by the DAS 28, the RADAI, HAQ questionnaires, the SF 36 (short health survey), response criteria number of clinical tests. Furthermore diverse analogue scales will be used to asses disease severity and pain in order to calculate the ACR response criteria.

Employment

At baseline, 28 weeks and 52 weeks questionnaires will be used to assess if adalimumab treatment has any influence on employment status, sickleave and workingcapacity (e.g.100 % fulltime or 50% parttime).

Anti-adalimumab T cells

In order to analyse the presence of an anti-adalimumab specific T cell compartment and map the specific T helper epitopes in the adalimumab sequence, we will apply distinct computer algorithms to predict epitopes against which T cell reactivity may be induced. The predicted epitopes will be analyzed in the context of the patients HLA profile and on average 25 peptides will be analyzed per patient. We will measure T cell proliferation against the predicted epitopes *in vitro* using CFSE labeling and FACS analyses.

In a subset of patients (n=20 anti-adalimumab antibody formers, n=40 non anti-adalimumab antibody formers and n=40 adalimumab naïve patients) 45ml (5 tubes) of heparinized blood at one time point. will be collected to isolate PBMC’s by ficoll gradient centrifugation. The cells will be labeled with the fluorochrome CFSE and cultured for 7 and 14days in the presence of the predicted peptides. A separate informed consent will be asked for this subgroup of patients.

Data analysis

We will describe the proportion of patients responding to adalimumab treatment who are TNF-α blockade naïve and patients who lacked response to prior anti-TNF-α treatment in relation to laboratory parameters. We will report clinical parameters of arthritis activity as well as markers of bone and cartilage destruction. Logistic regression modeling will be performed to identify distinguishing or predictive factors of response to TNF-α blockade among the clinical features at baseline and patient characteristics.

# 4. Ethical and legal aspects

## Good clinical practice

The procedures set out in this study protocol are designed to ensure that the investigator abide by the principles of the good clinical practice guidelines of the European Community and the Declaration of Helsinki *(see appendix)* in the conduct, evaluation and documentation of this study. The study will also be carried out in keeping with local legal requirements.

## Informed consent of patient

Before being admitted to the clinical study, the patient must consent to participate after the nature, scope and possible con­sequences of the clinical study have been explained in a form understandable to him/her. The patient will also be given an information sheet about the study and a copy of the consent form. The terms of the consent and when it was obtained must also be documented in the case record form.

In addition to the standard requirements that physicians are cur­rently obliged to observe when providing information, all points on the information sheet must be covered. The patient will give consent in writing. The signature of the investigator and patient or a witness must confirm the patient’s consent.

The investigator will not undertake any investigation specifically required only for the clinical study until valid consent has been obtained.

Approval of study protocol

Before the start of the study, the study protocol and/or other appropriate documents will be submitted to the ethics committee and/or the authori­ties, in accordance with local legal require­­ments.

Confidentiality

All patient names will be kept secret. The number al­lotted to them during the study will identify patients throughout documentation and evaluation. The patients will be told that all study findings will be stored on computer and handled in strictest confidence.

The signed informed consent forms remain with the investigator. The investi­gator agrees to obtain a correctly completed informed consent form for each patient included in the study. He/she also agrees to allow these to be inspected on request. The investiga­tor will maintain a personal list of patient numbers and patient names to enable records to be found at a later date. Both the consent forms and the personal list of patient numbers will be kept for 15 years.

## Liability and insurance

The civil liability of the investigator, the persons instructed by him and the hospital, practice or institute in which they are employed and the li­ability of AMC Medical Research B.V. (AMR) in respect of financial loss due to personal injury and other damage which may arise as a result of the carrying out of this study are governed by the applicable law.

The AMR has taken out reasonable third-party liability insurance cover. As a precautionary measure, the investigator, the persons instructed by him and the hospital, practice or institute are included in such cover in respect of work done by them in carrying out this trial to the extent that the claims are not covered by their own professional indemnity insur­ance.

Such insurance covers claims provided that:

the instructions and procedures laid down in the study protocol are followed precisely and the investigator acts with the necessary care

the patients do not undergo any other medical treatment without the previous consent of the investigator and that the investigator has in­formed patients to that effect

the patient informs the investigator immediately of any unforeseeable adverse events

the physical injury or other damage does not arise as a result of any gross negligence, willful act or omission

Use of study findings

By signing the study protocol, the investigator agrees with the content of the protocol , with the use of re­sults of the study for the purposes of national and international registra­tion and publication and information for medical and pharmaceutical professionals. If necessary, the authorities will be notified of the investigator­'s name, address, qualifications and extent of involvement.

The findings of this study may be published in a scientific jour­nal and pre­sented at scientific meetings.

# 5. Protocol amendments

The medical ethics committee must be informed of all amendments and if necessary approval must be sought for ethical aspects. Approval must also be obtained from the authorities if necessary.

6. Reference List

(1) Burmester GR, Stuhlmuller B, Keyszer G, Kinne RW. Mononuclear phagocytes and rheumatoid synovitis. Mastermind or workhorse in arthritis? Arthritis Rheum 1997; 40:5-18.

(2) Smeets TJ, Kraan MC, Galjaard S, Youssef PP, Smith MD, Tak PP. Analysis of the cell infiltrate and expression of matrix metalloproteinases and granzyme B in paired synovial biopsy specimens from the cartilage-pannus junction in patients with RA. Ann Rheum Dis 2001; 60(6):561-565.

(3) Tak PP, Smeets TJM, Daha MR, Kluin PM, Meijers KAE, Brand R et al. Analysis of the synovial cellular infiltrate in early rheumatoid synovial tissue in relation to local disease activity. Arthritis Rheum 1997; 40:217-225.

(4) Mulherin D, FitzGerald O, Bresnihan B. Synovial tissue macrophage populations and articular damage in rheumatoid arthritis. Arthritis Rheum 1996; 39:115-124.

(5) Elliott MJ, Maini RN, Feldmann M, Kalden JR, Antoni C, Smolen JS et al. Randomised double-blind comparison of chimeric monoclonal antibody to tumour necrosis factor alpha (cA2) versus placebo in rheumatoid arthritis. Lancet 1994; 344:1105-1110.

(6) Moreland LW, Baumgartner SW, Schiff MH, Tindall EA, Fleischmann RM, Weaver AL et al. Treatment of rheumatoid arthritis with a recombinant human tumor necrosis factor receptor (p75)-Fc fusion protein. N Engl J Med 1997; 337:141-147.

(7) Lipsky PE, van der Heijde DM, St Clair EW, Furst DE, Breedveld FC, Kalden JR et al. Infliximab and Methotrexate in the Treatment of Rheumatoid Arthritis. N Engl J Med 2000; 343(22):1594-1602.

(8) den Broeder A, van de PL, Rau R, Schattenkirchner M, Van Riel P, Sander O et al. A single dose, placebo controlled study of the fully human anti-tumor necrosis factor-alpha antibody adalimumab (D2E7) in patients with rheumatoid arthritis. J Rheumatol 2002; 29(11):2288-2298.

(9) Weinblatt ME, Keystone EC, Furst DE, Moreland LW, Weisman MH, Birbara CA et al. Adalimumab, a fully human anti-tumor necrosis factor alpha monoclonal antibody, for the treatment of rheumatoid arthritis in patients taking concomitant methotrexate: the ARMADA trial. Arthritis Rheum 2003; 48(1):35-45.

(10) Wolbink GJ, de Groot E, Lems W, Dijkmans B, Nurmohamed M, Tak PP, Voskuyl AE, Aarden LA. Anti-infliximab antibodies in patients with an infusion related allergic response to infliximab. Arthritis Rheum. 48, S326. 2003.

(11) Felson DT, Anderson JJ, Boers M, Bombardier C, Furst D, Goldsmith C et al. American College of Rheumatology preliminary definition of improvement in rheumatoid arthritis. Arthritis Rheum 1995; 38:727-735.

(12) Prevoo ML, 't Hof MA, Kuper HH, van Leeuwen MA, van de Putte LB, van Riel PL. Modified disease activity scores that include twenty-eight-joint counts. Development and validation in a prospective longitudinal study of patients with rheumatoid arthritis [see comments]. Arthritis Rheum 1995; 38(1):44-48.

13) Maini ME, Kremer JM, Bankhurst AD, Bulpitt KJ, Fleischman RM, Fox RI, Jackson CG, Lange M, Burge DJ. A trial of etanercept, a recombinant Tumor Necrosis Factor Receptor: Fc fusion protein, in patients with rheumatoid arthritis receiving methotrexate. New England Journal of Medicine 1999;340 (4) 253-259

14) **Bartelds** GM, **Wijbrandts** CA, **Nurmohamed**, MT, **Stapel S**, **Lems** WF, **Aarden** L, **Dijkmans BA**, **Tak PP**, **Wolbink** GJ. Clinical response to adalimumab: relationship to anti-adalimumab antibodies and serum adalimumab concentrations in rheumatoid arthritis. Ann Rheum Dis 2007; 66: 925-926

# Appendix I Classification of Functional Status

**Criteria for Classification of Functional Status in Rheumatoid Arthritis**

Class I Completely able to perform usual activities of daily living (self-

care, vocational, and avocational)

Class II Able to perform usual self-care and vocational actvities, but

limited in avocational activities

Class III Able to perform usual self-care actvities, but limited in

vocational avocational activities

Class IV Limited in ability to perform usual self-care, vocational and

avocational activities

Usual self-care activities include dressing, feeding, bathing, grooming, and toileting.

Avocational (recreational and/or leisure) and vocational (work, school, homemaking) activities are patient-desired and age- and sex specific.

The American College of Rheumatology 1991 revised criteria for the classification of global functional status in rheumatoid arthritis. *Arthritis Rheumatism 1992;35:498-502*

# Appendix II Declaration of Helsinki

WORLD MEDICAL ASSOCIATION DECLARATION OF HELSINKI

Ethical Principles forMedical Research Involving Human Subjects

Adopted by the 18^th^ WMA General Assembly, Helsinki, Finland, June 1964, and amended by the
29th WMA General Assembly, Tokyo, Japan, October 1975
35th WMA General Assembly, Venice, Italy, October 1983
41st WMA General Assembly, Hong Kong, September 1989
48th WMA General Assembly, Somerset West, Republic of South Africa, October 1996
and the 52nd WMA General Assembly, Edinburgh, Scotland, October 2000
Note of Clarification on Paragraph 29 added by the WMA General Assembly, Washington 2002

1. INTRODUCTION
   1. The World Medical Association has developed the Declaration of Helsinki as a statement of ethical principles to provide guidance to physicians and other participants in medical research involving human subjects. Medical research involving human subjects includes research on identifiable human material or identifiable data.
   2. It is the duty of the physician to promote and safeguard the health of the people. The physician's knowledge and conscience are dedicated to the fulfillment of this duty.
   3. The Declaration of Geneva of the World Medical Association binds the physician with the words, "The health of my patient will be my first consideration," and the International Code of Medical Ethics declares that, "A physician shall act only in the patient's interest when providing medical care which might have the effect of weakening the physical and mental condition of the patient."
   4. Medical progress is based on research which ultimately must rest in part on experimentation involving human subjects.
   5. In medical research on human subjects, considerations related to the well-being of the human subject should take precedence over the interests of science and society.
   6. The primary purpose of medical research involving human subjects is to improve prophylactic, diagnostic and therapeutic procedures and the understanding of the aetiology and pathogenesis of disease. Even the best proven prophylactic, diagnostic, and therapeutic methods must continuously be challenged through research for their effectiveness, efficiency, accessibility and quality.
   7. In current medical practice and in medical research, most prophylactic, diagnostic and therapeutic procedures involve risks and burdens.
   8. Medical research is subject to ethical standards that promote respect for all human beings and protect their health and rights. Some research populations are vulnerable and need special protection. The particular needs of the economically and medically disadvantaged must be recognized. Special attention is also required for those who cannot give or refuse consent for themselves, for those who may be subject to giving consent under duress, for those who will not benefit personally from the research and for those for whom the research is combined with care.
   9. Research Investigators should be aware of the ethical, legal and regulatory requirements for research on human subjects in their own countries as well as applicable international requirements. No national ethical, legal or regulatory requirement should be allowed to reduce or eliminate any of the protections for human subjects set forth in this Declaration.
2. BASIC PRINCIPLES FOR ALL MEDICAL RESEARCH
   1. It is the duty of the physician in medical research to protect the life, health, privacy, and dignity of the human subject.
   2. Medical research involving human subjects must conform to generally accepted scientific principles, be based on a thorough knowledge of the scientific literature, other relevant sources of information, and on adequate laboratory and, where appropriate, animal experimentation.
   3. Appropriate caution must be exercised in the conduct of research which may affect the environment, and the welfare of animals used for research must be respected.
   4. The design and performance of each experimental procedure involving human subjects should be clearly formulated in an experimental protocol. This protocol should be submitted for consideration, comment, guidance, and where appropriate, approval to a specially appointed ethical review committee, which must be independent of the investigator, the sponsor or any other kind of undue influence. This independent committee should be in conformity with the laws and regulations of the country in which the research experiment is performed. The committee has the right to monitor ongoing trials. The researcher has the obligation to provide monitoring information to the committee, especially any serious adverse events. The researcher should also submit to the committee, for review, information regarding funding, sponsors, institutional affiliations, other potential conflicts of interest and incentives for subjects.
   5. The research protocol should always contain a statement of the ethical considerations involved and should indicate that there is compliance with the principles enunciated in this Declaration.
   6. Medical research involving human subjects should be conducted only by scientifically qualified persons and under the supervision of a clinically competent medical person. The responsibility for the human subject must always rest with a medically qualified person and never rest on the subject of the research, even though the subject has given consent.
   7. Every medical research project involving human subjects should be preceded by careful assessment of predictable risks and burdens in comparison with foreseeable benefits to the subject or to others. This does not preclude the participation of healthy volunteers in medical research. The design of all studies should be publicly available.
   8. Physicians should abstain from engaging in research projects involving human subjects unless they are confident that the risks involved have been adequately assessed and can be satisfactorily managed. Physicians should cease any investigation if the risks are found to outweigh the potential benefits or if there is conclusive proof of positive and beneficial results.
   9. Medical research involving human subjects should only be conducted if the importance of the objective outweighs the inherent risks and burdens to the subject. This is especially important when the human subjects are healthy volunteers.
   10. Medical research is only justified if there is a reasonable likelihood that the populations in which the research is carried out stand to benefit from the results of the research.
   11. The subjects must be volunteers and informed participants in the research project.
   12. The right of research subjects to safeguard their integrity must always be respected. Every precaution should be taken to respect the privacy of the subject, the confidentiality of the patient's information and to minimize the impact of the study on the subject's physical and mental integrity and on the personality of the subject.
   13. In any research on human beings, each potential subject must be adequately informed of the aims, methods, sources of funding, any possible conflicts of interest, institutional affiliations of the researcher, the anticipated benefits and potential risks of the study and the discomfort it may entail. The subject should be informed of the right to abstain from participation in the study or to withdraw consent to participate at any time without reprisal. After ensuring that the subject has understood the information, the physician should then obtain the subject's freely-given informed consent, preferably in writing. If the consent cannot be obtained in writing, the non-written consent must be formally documented and witnessed.
   14. When obtaining informed consent for the research project the physician should be particularly cautious if the subject is in a dependent relationship with the physician or may consent under duress. In that case the informed consent should be obtained by a well-informed physician who is not engaged in the investigation and who is completely independent of this relationship.
   15. For a research subject who is legally incompetent, physically or mentally incapable of giving consent or is a legally incompetent minor, the investigator must obtain informed consent from the legally authorized representative in accordance with applicable law. These groups should not be included in research unless the research is necessary to promote the health of the population represented and this research cannot instead be performed on legally competent persons.
   16. When a subject deemed legally incompetent, such as a minor child, is able to give assent to decisions about participation in research, the investigator must obtain that assent in addition to the consent of the legally authorized representative.
   17. Research on individuals from whom it is not possible to obtain consent, including proxy or advance consent, should be done only if the physical/mental condition that prevents obtaining informed consent is a necessary characteristic of the research population. The specific reasons for involving research subjects with a condition that renders them unable to give informed consent should be stated in the experimental protocol for consideration and approval of the review committee. The protocol should state that consent to remain in the research should be obtained as soon as possible from the individual or a legally authorized surrogate.
   18. Both authors and publishers have ethical obligations. In publication of the results of research, the investigators are obliged to preserve the accuracy of the results. Negative as well as positive results should be published or otherwise publicly available. Sources of funding, institutional affiliations and any possible conflicts of interest should be declared in the publication. Reports of experimentation not in accordance with the principles laid down in this Declaration should not be accepted for publication.
3. ADDITIONAL PRINCIPLES FOR MEDICAL RESEARCH COMBINED WITH MEDICAL CARE
   1. The physician may combine medical research with medical care, only to the extent that the research is justified by its potential prophylactic, diagnostic or therapeutic value. When medical research is combined with medical care, additional standards apply to protect the patients who are research subjects.
   2. The benefits, risks, burdens and effectiveness of a new method should be tested against those of the best current prophylactic, diagnostic, and therapeutic methods. This does not exclude the use of placebo, or no treatment, in studies where no proven prophylactic, diagnostic or therapeutic method exists. [See footnote](http://www.wma.net/e/policy/#footnote)
   3. At the conclusion of the study, every patient entered into the study should be assured of access to the best proven prophylactic, diagnostic and therapeutic methods identified by the study.
   4. The physician should fully inform the patient which aspects of the care are related to the research. The refusal of a patient to participate in a study must never interfere with the patient-physician relationship.
   5. In the treatment of a patient, where proven prophylactic, diagnostic and therapeutic methods do not exist or have been ineffective, the physician, with informed consent from the patient, must be free to use unproven or new prophylactic, diagnostic and therapeutic measures, if in the physician's judgement it offers hope of saving life, re-establishing health or alleviating suffering. Where possible, these measures should be made the object of research, designed to evaluate their safety and efficacy. In all cases, new information should be recorded and, where appropriate, published. The other relevant guidelines of this Declaration should be followed.

**Footnote**: **Note of clarification on paragraph 29 of the WMA Declaration of Helsinki**

The WMA hereby reaffirms its position that extreme care must be taken in making use of a placebo-controlled trial and that in general this methodology should only be used in the absence of existing proven therapy. However, a placebo-controlled trial may be ethically acceptable, even if proven therapy is available, under the following circumstances:

  - Where for compelling and scientifically sound methodological reasons its use is necessary to determine the efficacy or safety of a prophylactic, diagnostic or therapeutic method; or

  - Where a prophylactic, diagnostic or therapeutic method is being investigated for a minor condition and the patients who receive placebo will not be subject to any additional risk of serious or irreversible harm.

All other provisions of the Declaration of Helsinki must be adhered to, especially the need for appropriate ethical and scientific review.

The Declaration of Helsinki (Document 17.C) is an official policy document of the World Medical Association, the global representative body for physicians. It was first adopted in 1964 (Helsinki, Finland) and revised in 1975 (Tokyo, Japan), 1983 (Venice, Italy), 1989 (Hong Kong), 1996 (Somerset-West, South Africa) and 2000 (Edinburgh, Scotland). Note of clarification on Paragraph 29 added by the WMA General Assembly, Washington 2002.

1. "Associated" means a reasonable possibility that the event may have been caused by the drug. If the [↑](#footnote-ref-2)
